# Trends in quality of non-Hodgkin’s lymphoma care: is it getting better?

**DOI:** 10.1007/s00277-015-2340-0

**Published:** 2015-03-15

**Authors:** J. J. C. Stienen, P. B. Ottevanger, L. Wennekes, S. A. M. van de Schans, H. M. Dekker, R. W. M. van der Maazen, J. H. J. M. van Krieken, N. M. A. Blijlevens, R. P. M. G. Hermens, On behalf of the PEARL study group

**Affiliations:** 1 Department of IQ Healthcare, Radboud University Medical Center, Radboud Institute for Health Sciences, PO box 9101, 6500 HB Nijmegen, The Netherlands; 2 Department of Medical Oncology, Radboud University Medical Center, Radboud Institute for Health Sciences, PO box 9101, 6500 HB Nijmegen, The Netherlands; 3 Department of Radiology, Radboud University Medical Center, Radboud Institute for Health Sciences, PO box 9101, 6500 HB Nijmegen, The Netherlands; 4 Department of Radiotherapy, Radboud University Medical Center, Radboud Institute for Health Sciences, PO box 9101, 6500 HB Nijmegen, The Netherlands; 5 Department of Pathology, Radboud University Medical Center, Radboud Institute for Health Sciences, PO box 9101, 6500 HB Nijmegen, The Netherlands; 6 Department of Hematology, Radboud University Medical Center, Radboud Institute for Health Sciences, PO box 9101, 6500 HB Nijmegen, The Netherlands; 7 Department of Registry and Research, Comprehensive Cancer Centre the Netherlands, PO box 19079, 3501 DB Utrecht, The Netherlands

**Keywords:** Non-Hodgkin’s lymphoma, Quality of health care, Guidelines, Oncology, Hematology, Transparency, Trends

## Abstract

This study outlines trends in quality of delivered non-Hodgkin’s lymphoma (NHL) care in the Netherlands between 2007 and 2011 and to what extend this was influenced by the national Visible Care program, which aimed at increasing transparency by providing insight into the quality of healthcare. We analyzed data collected from medical records in two observational studies, combined into 20 validated quality indicators (QIs) of which 6 were included in the national program. A random sample of 771 patients, diagnosed with NHL in 26 Dutch hospitals, was examined. Multilevel regression analyses were used to assess differences in quality of NHL care and to provide insight into the effect of the national program. We reported improved adherence to only 3 out of 6 QIs involved in the national program and none of the other 14 validated QIs. Improvement was shown for performance of all recommended staging techniques (from 26  to  43 %), assessment of International Prognostic Index (from 21  to  43 %), and multidisciplinary discussion of patients (from 23  to  41 %). We found limited improvement in quality of NHL care between 2007 and 2011; improvement potential (<80 % adherence) was still present for 13 QIs. The national program seems to have a small positive effect, but has not influenced all 20 indicators which represent the most important, measurable parts in quality of NHL care. These results illustrate the need for tailored implementation and quality improvement initiatives.

## Introduction

Non-Hodgkin’s lymphoma (NHL) affects over 300,000 people worldwide each year [1]. NHL is listed in the top 10 of most common cancers, with an estimated number of 69,000 new cases in the USA in 2013 [2] and responsible for 3 % of all cancer deaths in the USA [3]. In the Netherlands, the incidence of NHL is as high as 4000 newly diagnosed patients per year. With an expanding and ageing population, these figures are expected to increase. Well-organized and well-carried-out diagnostics and treatments are essential to help patients in the best possible way. Treatment of NHL is highly dependent on the subtype, stage, and aggressiveness of the tumor. For the most common subtypes, diffuse large B-cell lymphoma (DLBCL) and follicular lymphoma (FL), chemotherapy combined with immunotherapy (i.e., monoclonal antibody rituximab) has improved patient outcomes remarkably [4, 5]. Despite these improvements, the 5-year relative survival rate for these patients is only 50–75 % [6].

To deliver high-quality care to patients diagnosed with NHL, multidisciplinary evidence-based guidelines are developed to help professionals in their choices about diagnostics and therapy [7–10]. Quality of care can be measured using quality indicators, which are “measurable elements of practice performance for which there is evidence or consensus that they can be used to assess the quality of care” [11]. From previous research, it is known that discrepancies exist between NHL care delivered in daily practice and the (quality of) care recommended in guidelines [12–14]. Gaining insight into quality of delivered care is an upcoming phenomenon in healthcare; hospitals and other healthcare facilities are exposed to (external) auditing and benchmarking, often using quality indicators [15–17]. Especially healthcare insurances, policy makers (government) and patient organizations are important actors. The USA and UK have both introduced public reporting as a tool to improve the quality of care. National institutes as the National Quality Forum and the Dr Foster Health focus on delivering healthcare information to the public, for example, by providing a hospital guide to the public including information on waiting times, length of stay, and mortality rates for several surgical procedures [18].

In the Netherlands, a national initiative of transparency in hospital care started in 2008, raised and funded by the government (Visible Care program). The aim of this initiative was to increase transparency by providing insight into the quality of healthcare and enabling healthcare providers to report on actually applied diagnostics and delivered therapy and follow-up. Quality indicators were developed and measured for over 100 disease entities, including malignant lymphoma.

This study outlines the differences in quality of delivered NHL care in the Netherlands between 2007 and 2011 and to what extend this was influenced by the Visible Care program.

## Materials and methods

### Study design and population

Data from two observational studies were used to investigate the natural course of delivered quality of NHL care and the influence of the Visible Care program. First, Wennekes et al. developed quality indicators and measured their performance in 22 Dutch hospitals by including 348 patients diagnosed with NHL between 2006 and 2007 [14]. The quality indicators were derived from evidence-based guidelines and developed by an expert panel using the systematic RAND-modified Delphi method [19]. The main goal was to provide insight into guideline adherence for the most important processes and structures of NHL care. Second, baseline measurements of the Perineal Assessment and Repair Longitudinal (PEARL) study [20], a cluster randomized controlled trial (cRCT), also assessed indicator performance in 19 Dutch hospitals and randomly included 423 patients diagnosed with NHL between 2010 and 2011. Patients eligible for both studies were defined as patients diagnosed with a mature B-, T-, or NK-cell neoplasm and older than 18 years at diagnosis. Patients with multiple myeloma or cutaneous lymphoma or presenting with chronic leukemia were excluded.

### Data collection

For the PEARL study, trained registration employees collected data from medical records for the quality indicators. A digital registration form was accomplished in collaboration with the Comprehensive Cancer Centre the Netherlands (CCC). Selection of patients took place using the cancer registry, which is part of the CCC. The CCC used the cancer registry to make a list of potentially eligible patients in the participating hospitals. Patients were randomly listed, after which the first 30 patients were selected and data was collected for 20–25 patients per hospital. This cancer registry is based on the pathology coding system of the World Health Organization (WHO), and patients with mature B-, T-, and NK-cell neoplasms were selected for inclusion.

Patient characteristics (gender, age, hospital region, patient preferences, comorbidities) and tumor-specific information (morphology, stage, extra nodal disease, previous malignancies, and lactate dehydrogenase (LD) values) were collected. Furthermore, dates and data about diagnosis (pathology, imaging techniques, blood counts), treatment of NHL (type of therapy, response), and multidisciplinary consultation were assessed. This information was used to determine the indicator performance scores: the percentage of patients who received care as recommended in the guidelines. If the indicator scores where <90 %, improvement potential was considered to be present [21, 22]. Quality indicators for the domain diagnosis and staging, treatment and follow-up, and organization and coordination were included in the indicator set (see Table 2). The data collection method for the PEARL study (2011 dataset) is in line with the method described by Wennekes et al. [14] (2007 dataset).

### The national transparency initiative

In the context of the Visible Care program, a national transparency initiative to provide insight into the quality of hospital care, quality indicators for malignant lymphoma were developed in 2010 [23]. The purpose was to develop quality indicators which represent the most important parts of NHL care and could be easily measured by the hospitals themselves. All disciplines involved in NHL care were asked to provide delegates to participate in the development of the indicator set. The final indicator set included indicators about Ann Arbor staging and International Prognostic Index (IPI) scoring, performing all required staging techniques such as bone marrow biopsy and CT scans, assessing therapy response, arranging multidisciplinary consultations, and providing all diagnostic results within 4 weeks. The indicator set as developed by Wennekes et al. [14] was used as one of the references during the development of this Visible Care indicator set. The implementation of the quality indicators was mandatory, since the program was part of new national policy. Each year, hospitals were asked to provide the malignant lymphoma indicator results about the previous year. Yearly feedback was given to the hospitals by providing anonymous results of all hospitals outlined by the hospitals’ own results. All anonymous results were made publically available through the website www.zichtbarezorg.nl, with the intention that these feedback methods would lead to higher quality of overall NHL care. It was the hospitals’ own responsibility in which way the yearly audit was performed and the feedback was processed.

Wennekes et al. [14] collected data of hospital care before the introduction of the Visible Care program, and we repeated this assessment after the first 2 years of the program. In this way, it was possible to provide insight into the proposed positive effect of the national transparency initiative on the quality of delivered medical care.

### Statistical analyses

Descriptive statistics were performed to get insight into the patient characteristics of the two study populations. Quality indicator scores were assessed for both datasets (2007 and 2011) and expressed in percentages to describe current practice. Univariate analyses (*Χ* ^*2*^ tests) were used to test whether differences in patient characteristics and quality indicator scores were present between 2007 and 2011.

The relation between patient and tumor characteristics (e.g., gender, previous malignancies, and LD value) and differences in quality indicator scores between 2007 and 2011 was studied using univariate logistic regression analyses (*P* < 0.05). Factors often mentioned as reasons for non-adherence were also included: Charlson index, performance status, Ann Arbor stage, patient preferences, and type of lymphoma. Clinical relevant determinants that influenced quality indicator scores in a univariate setting were tested as a group using multivariate, multilevel logistic regression analyses (odds ratios). In case of intercorrelations between characteristics (>0.4), only one of the two was included in multivariate analyses. A multilevel model was used to account for the nested structure of the data, with individual patients nested within hospitals. The intraclass correlation coefficient (ICC) was calculated for each quality indicator to get insight into the cluster effect of the hospitals. ICC values between 0 and 0.40 are found in other secondary care research [24].

Reliability of the data collection was assessed with a duplicate registration of 30 records by two registration employees in three hospitals. The reliability of the indicator scores was calculated with a Kappa value, a statistical measure for interobserver agreement corrected for chance [25]. A missing values analysis (*t* test) was performed to explore if more than 5 % of the data was missing and if missing values were missing completely at random (Little’s test).

All statistical analyses, except multilevel analyses, were performed using IBM SPSS Statistics for Windows, version 20.0 (IMB Corp, Armonk, NY, USA). Multilevel analyses were performed using SAS software system for Windows, version 9.2 (SAS Institute Inc, Cary, NC, USA).

## Results

### Study population

Table 1 shows the characteristics of 348 patients of the 2007 dataset and 423 patients of the 2011 dataset. There were no significant differences in gender, age, prevalence of NHL subtypes, aggressiveness of the tumor, Charlson index, patient preferences, and hospital region between the two study populations. In 2011, more patients were included with previous malignancies, Ann Arbor stages III–IV, and normal LD values, compared to 2007.

**Table 1:** Patient characteristics in 2007 and 2011

Characteristics	2007 (*N* = 348)		2011 (*N* = 423)	
	%	95 % CI	%	95 % CI
Gender				
Male	56	51–61	57	52–62
Female	44	39–49	43	38–48
Missing		*N*=13		
Mean age (years)	66 ± 14		66 ± 13	
Charlson index^a^				
High risk	47	42–52	46	41–51
Low risk	53	48–58	54	49–59
Missing		*N* = 1		
NHL type				
DLBCL	42	37–47	46	41–51
Follicular lymphoma	23	19–27	18	14–22
Mantle-cell lymphoma	9	6–12	5	3–7
Marginal zone B-cell lymphoma	8	5–11	11	8–14
Other classification	18	14–22	20	16–24
Tumor type				
Aggressive	61	56–66	60	55–65
Indolent	39	34–44	40	35–45
Ann Arbor stage*				
I or II	44	38–50	32	27–37
III or IV	56	50–62	68	63–73
Unknown		*N* = 2		*N* = 14
Not applicable		*N* = 39		*N* = 14
Extranodal disease				
Yes	58	52–64	61	56–66
No	42	36–48	39	34–44
Missing		*N* = 42		*N* = 1
Previous malignancies*				
No (NHL is first malignancy)	91	88–94	83	79–87
Yes	9	6–12	17	13–21
Missing		*N* = 1		
Normal LD value*				
Yes	20	16–24	56	51–61
No	80	76–84	44	39–49
Missing				*N* = 32
Patient’s preferences				
Objections	6	4–8	6	4–8
No objections	94	92–96	94	92–96
Missing		*N* = 1		*N* = 1
Hospital region				
North	53	48–58	45	40–50
East	25	20–30	27	23–31
South	22	18–26	28	24–32

*DLBCL* diffuse large B-cell lymphoma, *LD* lactate dehydrogenase

*Patient characteristic significantly different (*P* < 0.05) between 2007 and 2011

^a^Weighted comorbidity index combined with age

### Quality indicator scores

Table 2 describes the 20 quality indicator scores, corrected for case-mix (i.e., gender, Ann Arbor stage, aggressiveness, previous malignancies, LD value, Charlson index, and hospital region) and the nested structure of the data (multilevel analysis). The quality indicators were divided into three domains: diagnosis and staging, treatment and follow-up, and organization and coordination of care.

**Table 2:** Indicator scores for quality of delivered NHL care in 2007 and 2011

Quality indicator	% 2007	Number of samples	Missing	Excluded	% 2011	Number of samples	Missing	Excluded	OR year (95 % CI)^a^
Diagnosis and staging									
QI 1 Diagnosis based on histological examination and on an excision or wide incision biopsy	82	212	93	43	79	369	21	33	0.840 (0.494–1.428)
A. Histological examination	97	305	0	43	97	389	1	33	
B. Excision or wide incision biopsy^b^	82	212	93	43	80	369	21	33	
QI 2 Patients staged according to the Ann Arbor classification	80	305	0	43	81	390	0	33	1.102 (0.705–1.724)
QI 3 Diagnosis for NHL based on morphology and immune phenotype	99	283	23	43	96	376	14	33	*0.190 (0.049–0.744)*
A. Morphology	100	287	18	43	100	378	12	33	
B. Immune phenotype	99	291	16	43	97	378	12	33	
C. No molecular clonality (or only supplementary)	100	284	21	43	99	376	14	33	
QI 4 Staging techniques should include CT scans of the neck, thorax, and abdomen, bone marrow aspirate, and bone marrow biopsy	26	347	1	0	43	421	2	0	**1.902 (1.268–2.852)**
A. CT scan of the neck	41	348	0	0	46	421	2	0	
B. CT scan of the thorax	84	348	0	0	75	421	2	0	
C. CT scan of the abdomen	84	348	0	0	77	421	2	0	
D. Bone marrow aspirate	68	348	0	0	66	421	2	0	
E. Bone marrow biopsy (crista)	85	347	1	0	78	421	2	0	
QI 5 Assessment of International Prognostic Index (IPI) for patients with aggressive NHL	21	213	0	135	43	250	4	169	**2.883 (1.675–4.961)**
QI 6 Assessment of lactate dehydrogenase value	93	347	1	0	92	423	0	0	0.995 (0.534–1.855)
QI 7 Examination of blood counts	90	348	0	0	82	422	1	0	*0.576 (0.340–0.976)*
A. Leukocyte count	99	348	0	0	98	422	1	0	
B. Leukocyte differentiation	91	348	0	0	83	422	1	0	
C. Thrombocyte count	99	348	0	0	97	422	1	0	
D. Hemoglobin level	99	348	0	0	98	423	0	0	
Treatment and follow-up									
QI 8 Reporting of response to therapy using complete remission, partial remission, stable disease, progression, recurrence	65	231	25	92	70	318	1	104	1.318 (0.840–2.068)
QI 9 All target lesions documented in radiology report before therapy	64	227	3	118	67	344	3	76	0.917 (0.587–1.432)
A. Location of lesions reported	99	228	2	118	100	344	3	76	
B. Size of lesions reported	74	227	3	118	77	344	3	76	
C. Size of lesions reported in mm	64	227	3	118	67	344	3	76	
QI 10 All target lesions documented in radiology report after therapy	67	82	3	263	58	114	3	306	0.589 (0.275–1.264)
A. Location of lesions reported	98	84	1	263	98	116	1	306	
B. Size of lesions reported	68	82	3	263	70	115	2	306	
C. Size of lesions reported in mm	68	83	2	263	58	114	3	306	
QI 11 Evaluation after chemotherapy with CT scans (or PET), and for stage IV patients also with a bone marrow aspirate and biopsy	61	176	12	160	62	246	5	172	1.583 (0.939–2.668)
A. CT scan before therapy	94	203	0	145	97	269	2	152	
B. CT/PET scan after therapy	77	185	13	150	89	250	4	169	
C. Bone marrow aspirate evaluation^c^	46	68	2	278	39	122	0	301	
D. Bone marrow biopsy evaluation^c^	37	68	2	278	45	122	0	301	
QI 12 Patients with DLBCL received chemotherapy with RCHOP	77	144	1	203	78	194	0	229	1.223 (0.663–2.258)
QI 13 Dose of RCHOP was not reduced or reason for reduction was reported	81	80	31	237	82	111	42	270	0.998 (0.378–2.631)
Organization and coordination of care									
QI 14 Sending and receiving of unfixed biopsy material	41	285	20	43	41	321	69	33	0.657 (0.419–1.032)
QI 15 Integrated reporting of pathology techniques	88	284	21	43	89	365	25	33	1.389 (0.752–2.564)
QI 16 Pathology report should be complete	12	279	26	43	14	378	12	33	1.379 (0.792–2.403)
A. Origin of tissue	91	285	20	43	98	378	12	33	
B. Tissue characteristics	56	286	19	43	52	378	12	33	
C. Biopsy method	77	305	0	43	91	378	12	33	
D. Receipt of material	41	284	20	43	34	378	12	33	
E. Frozen tissue	27	284	21	43	21	378	12	33	
QI 17 Patients discussed in multidisciplinary consultations	23	346	2	0	41	422	1	0	**3.360 (2.007–5.624)**
QI 18 Results of pathology known before the start of treatment (incl. bone marrow)	73	256	0	92	83	317	2	104	1.384 (0.866–2.210)
QI 19 Diagnostic period of 4 weeks after the first visit to the hospital	56	343	5	0	49	420	3	0	0.857 (0.599–1.227)
QI 20 Start of therapy within 2 weeks after diagnostic period	55	234	22	92	54	313	6	104	1.263 (0.822–1.941)

***Bold*** ORs represent a significant increase in indicator score between the two years

***Italicized*** ORs represent a significant decrease in indicator score between the two years/measurements?

The underlined quality indicator (QI) numbers are included in the Visible Care program

^a^Reference year 2007, odds ratios corrected for case-mix and ICC

^b^Excluding fine needle aspiration (FNA)

^c^Only applicable to stage IV patients

#### Diagnosis and staging

In this domain, improvement is seen for two of the seven quality indicators. Indicator scores for execution of all recommended staging techniques (QI4) improved from 26 to 43 % (OR = 1.902 (1.268–2.852)), and assessment of the International Prognostic Index (QI5) improved from 21 to 43 % (OR = 2.883 (1.675–4.961)). Two indicators scored significantly lower in 2011 compared to 2007: diagnosis based on morphology and immune phenotype decreased from 99 to 96 % (QI3: OR = 0.190 (0.049–0.744)) and examination of blood counts decreased from 90 to 82 % (QI7: OR = 0.576 (0.340–0.976)).

#### Treatment and follow-up

No significant differences are seen for the six quality indicators in this domain.

#### Organization and coordination of care

In this domain, only one of the seven quality indicator scores significantly improved (QI17): more patients (41 %) were discussed in multidisciplinary consultations in 2011 compared to 2007 (23 %) (OR = 3.360 (2.007–5.624)).

### Influence of the national transparency initiative

The six quality indicators used as initial concept for the Visible Care quality indicators are underlined in Table 2. Significantly increased quality indicator scores were found for three of the six indicators incorporated in the transparency initiative: the use of all staging techniques increased from 26 to 43 %, the assessment of the IPI from 21 to 43 %, and patients discussed in multidisciplinary consultations from 23 to 41 % (QI4, QI5, and QI17, respectively).

The other three quality indicators included in the transparency initiative did not change significantly: 80 % of the patients was staged according to the Ann Arbor classification (QI2), 60 % had a correct evaluation after chemotherapy (QI11), and the maximum diagnostic period of 4 weeks was realized in about half of the patients (QI19). Two of the 14 quality indicators that were not included in the Visible Care program showed significantly decreased scores (QI3 and QI7).

### Statistics

The mean ICC was 0.14 (range 0.005–0.33), which was calculated for all quality indicators to account for clustering. The reproducibility of the data collected for the indicator scores in 2011 was good: the average kappa value was 0.8 (range 0.4–1.0; 87 % ≥0.6). This indicates an overall good agreement between the registration employees. For the 2007 dataset, reproducibility was also good [14]. Missing value analysis showed overall less than 5 % missing data in the overall dataset (2007 and 2011). The factor “performance status” was excluded from analysis because it was missing for most of the cases (80 %). Quality indicator 13 had 10 % missing values; however, these were missing completely at random (MCAR, *P* = 0.771).

## Discussion

In this study, we provided insight into the differences in quality of delivered NHL care in the Netherlands between 2007 and 2011, taking into account the Visible Care program. The data indicated that quality of delivered NHL care improved at a few points, but also showed a greater need for improvement for several quality indicators. Indicator scores that decreased significantly included assessment of immune phenotype for diagnosis and full examination of blood counts. However, significant improvement was shown for execution of all recommended staging techniques, assessment of the IPI, and multidisciplinary discussion of patients.

Quality indicators included in the Visible Care program concerning malignant lymphoma were developed and published in 2010. The main goal of this program was to increase transparency of the Dutch hospital care, for which publically available data were required. This could have been a major incentive for hospitals to meet the quality indicators and thus improve their NHL care (if necessary). It is noticeable that the quality indicators with increased scores (QI4, QI5, and QI17) between 2007 and 2011 were all included in the national initiative, which implies a positive effect of the program. However, other (local) initiatives or international study results were not taken into account in our analysis. For example, the added value of discussing all oncology patients during multidisciplinary consultations has been an increasing point of interest the past years, which could have influenced our results.

Quality indicator scores that significantly decreased between the two measurements included items about pathology diagnosis based on immune phenotype (QI3) and examination of leukocyte differentiation during blood analyses (QI7). Despite these decreases, the scores were still between 80 and 100 %, which indicates good performance and only a small improvement potential. Improvement potential is often assigned to indicator scores below 90 % [21, 22], since higher scores might not be feasible due to case-mix factors as high age, many comorbidities, and patient preferences. It can therefore be argued if the significantly decreased scores seen for the two quality indicators are clinically relevant.

Between 2007 and 2011, there were no large changes in guideline recommendations for NHL care in the Netherlands, besides the addition of a recommendation to use fluorodeoxyglucose-positron emission tomography (FDG-PET) scans for determining therapy response [26, 27]. Using CT scanning, location and size in millimeters should be documented, which is not necessary when a FDG-PET scan is done, since it is based on color intensity instead of size. This could possibly explain the decreasing trend observed in the quality indicator concerning lesions documented in radiology reports after therapy (QI10 67 to 58 %, not significant). Based on our datasets, we determined that FDG-PET (CT-)scans were used more often for evaluation after chemotherapy in 2011 (63 %) compared to 2007 (29 %, *P* < 0.001).

Another decreasing trend was seen providing all diagnostic results within 4 weeks after the first visit to the hospital (QI19 56 to 49 %, not significant). A factor that may play a role is referral of patients between and within hospitals, which increased from 51 % in 2007 to 64 % in 2011 (*P* < 0.001). A possible explanation for this might be the increasing burden on the healthcare system, caused by the growing needs of patients. Although this decreasing trend is not significant, and therefore subject to chance, providing all diagnostic results within 4 weeks might be worth arguing because of its clinical importance concerning prognosis and start of treatment for patients diagnosed with NHL. We believe that longer waiting times could result into poorer prognosis for patients, which is already known for patients with head and neck cancer [28]. Furthermore, short waiting times are valuable for the level of patient centeredness of hospitals, of which the importance has grown considerably.

To our knowledge, this is the first study that focused on providing insight into the differences in time in quality of delivered NHL care and evaluated the effect of a national transparency initiative. Besides the present study and Wennekes et al.’s [14], only a few other studies conducted research on quality of NHL care, focusing on parts of the care process such as staging and treatment [12, 13, 29], or follow-up care [30]. In line with our study, they showed that the quality of NHL care is not yet optimal, compared to the recommended care as described in evidence-based guidelines.

Much research has been conducted concerning public reporting, with widely varying results. However, the effect of performance data on improvement of quality of care is less often studied, and if so, mostly focuses on mortality rates [31]. A study by Lamb et al. [32] explored the 5-year impact of voluntary public reporting on physician groups and showed improvement for ambulatory care measures. Our results are in accordance with these studies; however, we were able to provide insight into the quality of care for several measures not included in the public reporting as well. This provided the unique opportunity to examine the effect of public reporting, as done within the Visible Care program, on total quality of NHL care.

Data collection for observational studies included was performed by independent, trained registration employees. In daily practice or public reporting programs, professionals themselves are often responsible for the data collection, which might introduce bias as they are involved as stakeholders. Our data collection showed also high reproducibility between the registration employees, indicating good competency and reliable data collection.

There are also some limitations to this study. Firstly, the hospitals included for data collection in 2011 are not completely identical to those included in 2007: 15 hospitals participated in both measurements, 11 hospitals either in 2007 or 2011. However, no distinct differences were found in indicator scores when the non-overlapping hospitals were left out of the analyses. Together with a relatively large study population (*N* = 771), we believe that both datasets are representative for hospitals in the Netherlands.

Secondly, we did not have insight into possible local improvement initiatives of the participating hospitals between 2007 and 2011, which makes it difficult to link the Visible Care program as direct and only attribute to the changes seen in some quality indicators of NHL care. One influencing factor could be that high room for improvement rates were seen for four of the six quality indicators included in this program (<30 % scores, 2007 dataset). It can be argued that, in general, quality indicators with low scores are easier to improve than those with higher scores. Hospitals, participated in the 2007 study, received general feedback on their performance as a group and as hospital region, which might have triggered some local improvement initiatives. However, improving NHL care was not the aim of the 2007 study, and no concrete improvement initiatives are known based on the provided feedback. This result is supported by a Cochrane review, which showed that the effect of audit and feedback on improvement is usually small [33].

In summary, during the 4-year period between 2007 and 2011, we found little improvement in overall quality of NHL care. Improvement potential as found in 2007 remained for all quality indicators in 2011, unless some significant increased indicator scores. Based on our results, we can conclude that a national initiative as the Visible Care program might not have enough power to improve hospital care for NHL in general, but might provide a first step towards a more improvement-oriented hospital care. Improvement strategies tailored to the suboptimal quality of NHL care might be a rational step to develop and test in a randomized setting.
